# Savanna tree evolutionary ages inform the reconstruction of the paleoenvironment of our hominin ancestors

**DOI:** 10.1038/s41598-020-69378-0

**Published:** 2020-07-24

**Authors:** T. Jonathan Davies, Barnabas H. Daru, Bezeng S. Bezeng, Tristan Charles-Dominique, Gareth P. Hempson, Ronny M. Kabongo, Olivier Maurin, A. Muthama Muasya, Michelle van der Bank, William J. Bond

**Affiliations:** 1 Department of Botany and Plant Biotechnology, African Centre for DNA Barcoding, University of Johannesburg, Johannesburg, 2006 South Africa; 2 Departments of Botany, Forest and Conservation Sciences, Biodiversity Research Centre, University of British Columbia, Vancouver, BC V6T, 1Z4 Canada; 3 Department of Life Sciences, Texas A&M University-Corpus Christi, Corpus Christi, TX 78412 USA; 4 Department of Biological Sciences, University of Cape Town, Rondebosch, 7701 South Africa; 5 Xishuangbanna Tropical Botanical Garden, Chinese Academy of Sciences, Center for Integrated Conservation, Community Ecology and Conservation, Menglun, 666303 Yunnan China; 6 School of Animal, Plant and Environmental Sciences, University of the Witwatersrand, Wits, 2050 South Africa; 7 South African Environmental Observation Network, National Research Foundation, Pretoria, 0083 South Africa; 8 Royal Botanic Gardens, Kew Richmond, Surrey, TW9 3DS UK

**Keywords:** Palaeoclimate, Biological anthropology, Biogeography, Ecosystem ecology, Phylogenetics

## Abstract

Ideas on hominin evolution have long invoked the emergence from forests into open habitats as generating selection for traits such as bipedalism and dietary shifts. Though controversial, the *savanna hypothesis* continues to motivate research into the palaeo-environments of Africa. Reconstruction of these ancient environments has depended heavily on carbon isotopic analysis of fossil bones and palaeosols. The sparsity of the fossil record, however, imposes a limit to the strength of inference that can be drawn from such data. Time-calibrated phylogenies offer an additional tool for dating the spread of savanna habitat. Here, using the evolutionary ages of African savanna trees, we suggest an initial tropical or subtropical expansion of savanna between 10 and 15 Ma, which then extended to higher latitudes, reaching southern Africa ca. 3 Ma. Our phylogenetic estimates of the origin and latitudinal spread of savannas broadly correspond with isotopic age estimates and encompass the entire hominin fossil record. Our results are consistent with the savanna hypothesis of early hominin evolution and reignite the debate on the drivers of savanna expansion. Our analysis demonstrates the utility of phylogenetic proxies for dating major ecological transitions in geological time, especially in regions where fossils are rare or absent or occur in discontinuous sediments.

## Introduction

The emergence of savannas and other tropical grassy biomes has been a topic of intense research interest, not least because it coincides with early hominin evolution. The *savanna hypothesis* of human evolution suggests that the transition from a predominately arboreal lifestyle in forest to one in open habitats favoured an upright posture and selected for bipedalism along with a shift in diet that necessitated travel over greater distances across the landscape^1^. The early support for the *savanna hypothesis* waned, in part, due to confusion as to the definition of prehistoric savannas—as open grassland or as a grassland-tree mosaic—nonetheless, it continues to influence thinking about the selective landscape that shaped human evolution, and generate large interest in the palaeo-environments of Africa where our ancestors emerged^2–6^. However, while our understanding of hominin evolution is continually updated by new fossil finds, the palaeontological reconstruction of the ancient African environment has been greatly limited by the sparse record of fossil bones and palaeosols that capture the signature of these past ecosystems^7^.

The origin and spread of savannas is thought to be closely linked to the attributes of the C_4_ grasses that dominate the herbaceous layer^8,9^. An influential hypothesis for the spread of the savanna biome suggests that C_4_ grasses likely first evolved when atmospheric CO_2_ decreased below a threshold of 500 ppm, which was thought to have occurred in the Late Miocene, appearing first at the equator with warm growing season temperatures and progressively moving to higher latitudes with cooler climates^10,11^. Low CO_2_ concentrations and high temperatures during the growing season would have favoured the C_4_ photosynthetic pathway, a CO_2_ concentrating mechanism, allowing C_4_ grasses to thrive relative to C_3_ plants^11^. Subsequent studies suggested that *p*CO_2_ fell below the threshold favouring C_4_ photosynthesis in the Oligocene, leading to rejection of the physiological model for a late Miocene origin of savannas^12–14^. However, new proxies support a steep decline in *p*CO_2_ from ~ 7 Ma^15,16^ which again raises the question of whether the timing of savanna origins along a latitudinal gradient is rooted in C_3_ versus C_4_ photosynthetic physiology.

Much of the evidence for a shift from forests to savannas comes from analyses of carbon isotopes in fossil soils and fossil bones. For example, it is possible to estimate tree cover in savannas from the ratio of C^13^ to C^12^ in current and fossil soil carbon^2^. Similarly, the dietary mix of fossil hominins can be traced from carbon isotope analysis^17^. Other proxies for reconstructing ancient habitats include pollen, alkanes, and rates of dust deposition into lakes or marine cores^3,5,6,18^. Together, these proxies are providing increasingly detailed reconstructions of environmental conditions and their variability over the past few million years. However, sites with suitable temporal continuity are few and largely restricted to East Africa. Fossil sites elsewhere in Africa are patchily distributed in space and time, and therefore provide only snapshots of environmental conditions in the past^3,19^.

Dated phylogenetic trees reconstructed using molecular sequence data and calibrated from the fossil record, offer an alternative source of information, and insights deeper in time. For example, the phylogenetic structure of modern species assemblages can reveal insights into historical biogeography, while the timing of lineage diversification and evolutionary radiations may be linked to shifts in dominant habitat types and climates^20,21^. New ecological opportunity brought about by dispersal to a new environment, the acquisition of a key innovation, or extinction of a competitor, might trigger adaptive radiations. The appearance of new clades sharing a particular adaptive trait can therefore capture important ecological shifts that might not be detectable in the fossil record^22,23^.

It would seem reasonable to use phylogenetic methods to date the origin of savannas by dating the origin of C_4_ grasses from C_3_ ancestors, yet phylogenetic analyses of C_4_ grasses^24^ have placed their evolutionary origin in the Oligocene, ca. 30 Ma ago, and more than 20 Ma before isotopic evidence supporting the spread of savannas^10,19^. Diverse proxies indicate a steep decline in CO_2_ in the early Oligocene^12^, consistent with the physiological arguments for the advantages of the C_4_ CO_2_ concentration mechanism. However, environmental conditions favouring selection for C_4_ photosynthesis were not the same as those favouring the assembly and spread of C_4_ grassy biomes^25^: the evolution of the C_4_ pathway was a necessary but not sufficient prerequisite. The C_4_ pathway appears to have been a preadaptation to the more seasonal, open environments which were to emerge later. The phylogeny of the grasses that define the biome does not, therefore, allow us to date the key ecological transition from closed forests to open grassy ecosystems.

Rather than focus on the age of taxa that define a habitat, the dating of lineages that have diversified within that habitat might better capture information on its origins. For example, epiphytes or lianas, rather than trees, can be used as markers of closed forest: trees create the closed canopy, but epiphytes and lianas that are restricted to forest, provide a much better indicator of the presence and extent of forest habitat^26^. Predicated on the reasonable assumption that speciation rates correlate with available area^27^, lineage diversification within a habitat may provide an indicator of its geographical extent. Thus, by dating the radiation of clades tightly associated with a particular biome, we may infer its likely age^28^. Knowledge on the ecology of radiating clades can additionally provide insights into the environmental context that favoured important ecological and evolutionary transitions. For example, in the Cerrado, a South American C_4_ savanna, the evolution of fire-resistance arose multiple times independently within the last 4 to 10 Ma, and suggests a relatively recent, fiery, origin of Cerrado vegetation^29^. Here we use the evolutionary relationships between savanna and forest trees to explore the origins of African savanna.

## Results

Using a well-sampled, time-calibrated phylogenetic tree of the woody flora, we examine the timing of 139 evolutionary splits between savanna trees in Africa as a marker for the age of the savanna biome. Because we are interested in capturing the oldest diversifications, as these will most closely align to the age of the biome, we focus on these older dates. We then project the distribution of branching times on to a map of southern Africa to explore the geographic arrangement in the timing of expansion of the savanna biome across latitudes.

**Figure 1:**
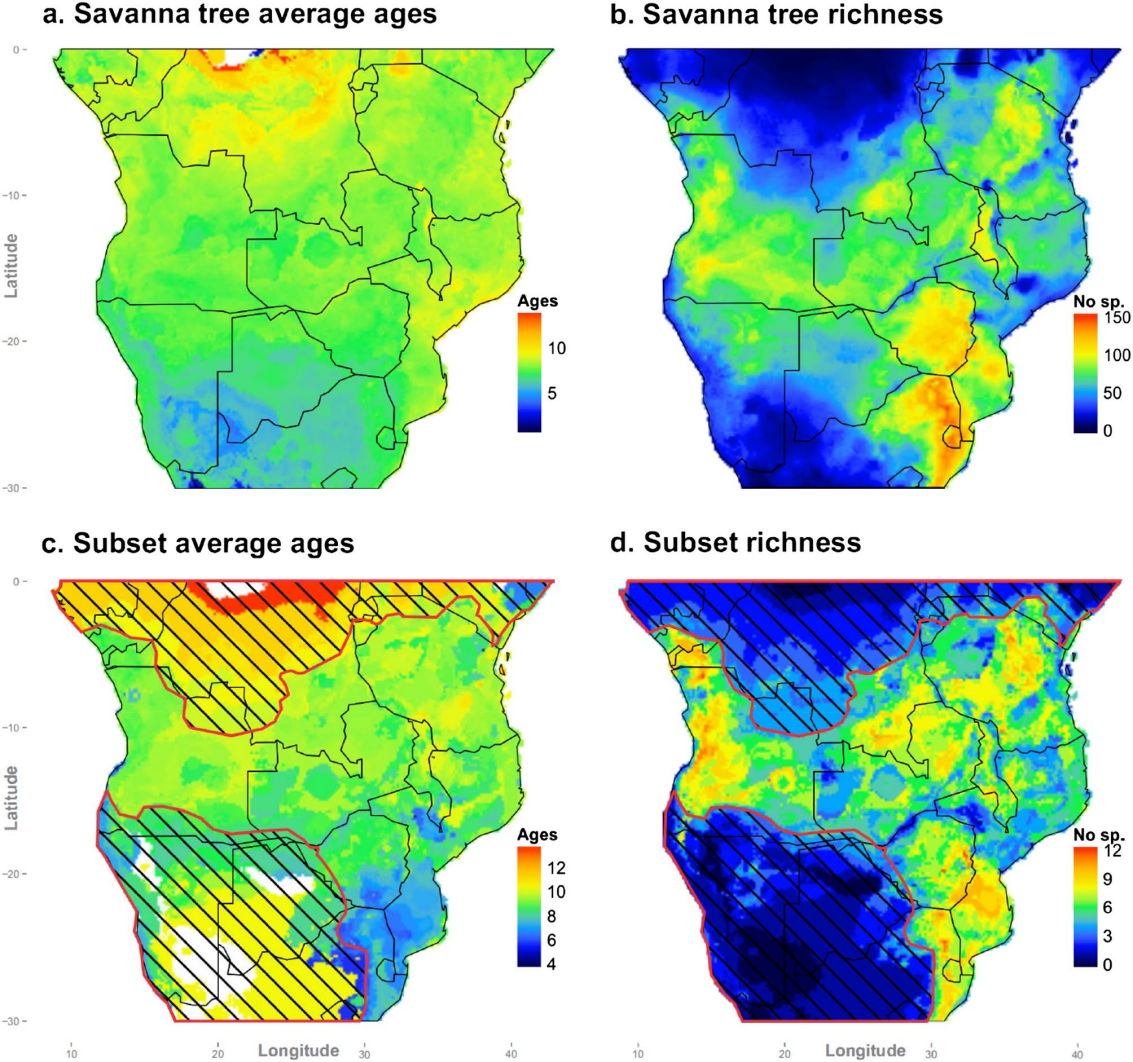
Geographical distribution of splits between savanna sister tree species. (**a)**, distribution of mean ages in millions of years for savanna tree evolutionary splits (Supplementary Table 1); (**b**), species richness of savanna trees; (**c**), distribution of savanna tree sister ages estimated from the 90th quantile of evolutionary splits fit to the regression in Fig. 2; and (**d)**, richness of savanna tree species used for the estimation in (**c**). Hatched areas correspond to low diversity regions not supporting savanna vegetation (in the south) and under-sampling in the north. Maps were generated in the R statistical computing environment (version 3.2.0; https://www.r-project.org/) using the R library ggplot2 (version 1.0.1; https://ggplot2.tidyverse.org/).

**Figure 2:**
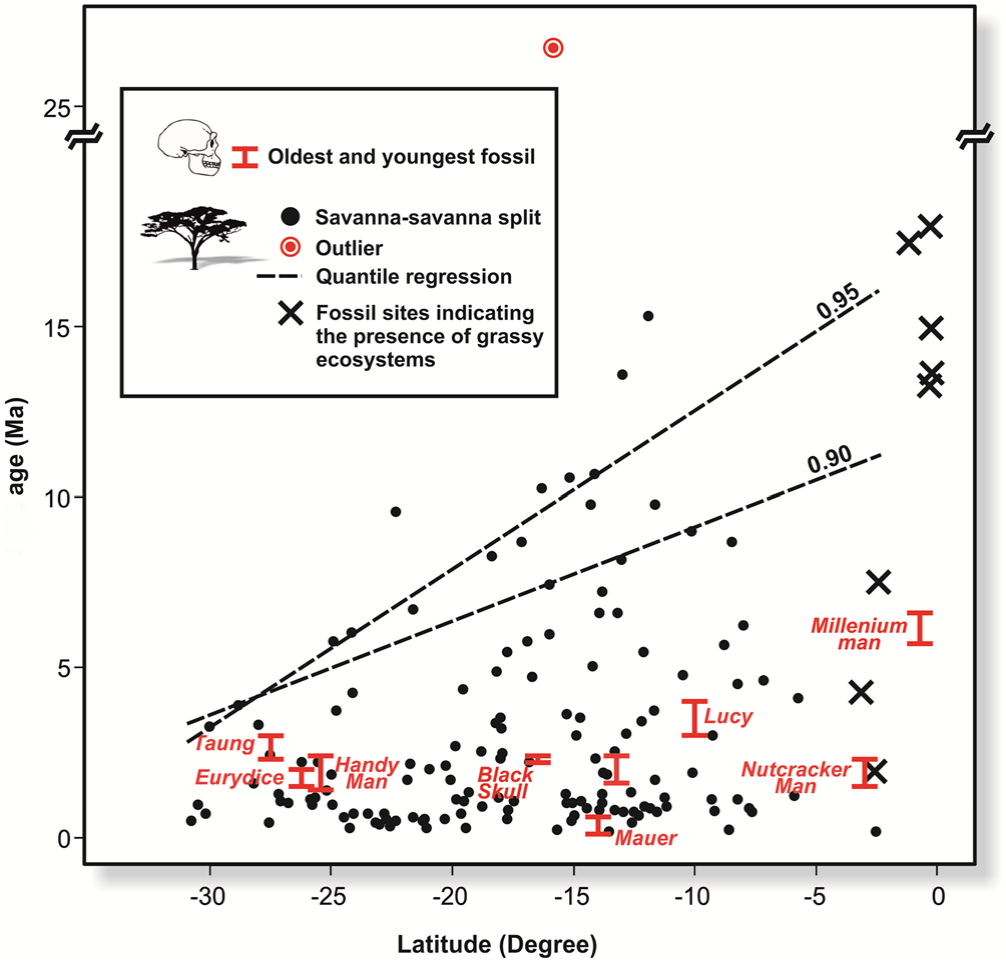
Quantile regression of savanna tree evolutionary splits across latitudes. The dashed lines indicate the 90th and 95th quantiles of divergence times between savanna trees (see “Methods” section), and is referenced to the age of hominin fossils at equivalent latitudes extracted from the literature (Supplementary Table 2). Latitude indicates degrees south. The age and latitude of fossil sites indicating the presence of grassy ecosystems (data from ref.^32^ and included in Supplementary Table 3) shown with X’s, and highlight the sparsity of fossil data at higher latitudes.

Our results provide unique phylogenetic evidence supporting a latitudinal gradient in the spread of savanna across Africa (Fig. 1). The phylogenetic signature of savanna expansion indicates a southerly progression from more tropical latitudes over several millions of years (slope = 0.28 [s.e. = 0.11], t = 2.59, *p* < 0.02, from the quantile linear regression on the top 10% oldest savanna tree ages; Fig. 2). One obvious outlier (see Fig. 2), representing the evolutionary split between *Bolusanthus speciosus* and *Pericopsis angolensis* within Fabaceae, was excluded from the quantile regression. Divergence times for this sister pair were likely overestimated because of the relatively poor sampling of closely related species within these clades (see ref.^30^). In contrast, we observed no obvious gradient in divergence times across latitude for forest trees (slope = 0.08 [s.e. = 0.14], t = 0.55, *p* = 0.58, from the quantile linear regression on the top 10% oldest forest tree ages; see Supplementary Figure S1, which shows the latitudinal trend in evolutionary splits in forest sister taxa, excluding divergences older than 25 Ma for comparison with Fig. 2). If the latitudinal trend indicating younger ages at higher latitudes was simply a sampling artefact (e.g. a greater sampling of taxa at high latitudes more finely splitting evolutionary branch lengths separating sister species), such bias should be equally apparent in forest and savanna trees. The quantile regression through the top 5% oldest ages reveals a similar distinction between latitudinal trends in forest and savanna tree ages (slope = 0.46 [s.e. = 0.14], t = 3.38, *p* < 0.01 and slope = 0.18 [s.e. = 0.21], t = 0.86, *p* = 0.39, for savanna and forest trees, respectively), and suggests an older age of savanna trees at the equator, although this estimate is informed by only few divergence times.

In Supplementary Figures S2 and S3 we show data for savanna and forest sister taxa, respectively, calculating mean latitude from ranges truncated at 15° North and excluding evolutionary splits between sister taxa older than 25 Ma (see above). Results are qualitatively similar to those truncating species distributions at the equator, supporting a strong latitudinal gradient in evolutionary splits between savanna trees, with older ages at lower latitudes, but little evidence for any latitudinal trend among forest species.

We found biome membership was significantly phylogenetically conserved (D = 0.59, and D = 0.57, for forest and savanna biome membership, respectively, where D = 1 indicates random phylogenetic structure and D = 0 matches to a Brownian motion model of trait evolution^31^, indicating that evolutionary transitions between biome type are relatively rare. Thus even if biomes shifted geographically, the composition of lineages that comprise them may have changed little.

Our analysis indicates a best estimate of tropical savanna age of ca. 10–15 Ma, while at 30° S the savanna biome may date back to only ca. 3 Ma. Nonetheless, we recognise that there is uncertainty in our age estimates, reflecting phylogenetic uncertainty in phylogenetic node calibrations. Supplementary Figures S4 and S5 show the 95% Highest Posterior Density (HPD) intervals for divergence times between savanna trees (from ranges truncated at the equator and 15° North, respectively). Fitted lines are through the 10% quantile of the mean HPD (black) upper 95% HPD (red) and lower 5% HPD (blue). These figures suggest that tropical savanna could be as old as 20 + Ma, and as young as ~ 1–2 Ma. The latter estimate is highly implausible given current knowledge of the fossil record, and both upper and lower extremes do not reflect the true distribution of evolutionary splits represented by any single phylogenetic reconstruction (i.e. no one reconstructed phylogeny encompasses the plotted variation in evolutionary splits), but rather sample the maximum and minimum estimates of evolutionary splits between sister taxa across the entire posterior distribution of dated phylogenies, and we present them here simply to bracket the bounds of age estimates, and to illustrate the robustness of the latitudinal gradient in savanna age.

## Discussion

Using evolutionary divergence times between savanna trees, we infer a latitudinal gradient in the age of the savanna biome in Africa. Our best estimates of subtropical savanna ages suggest a date of biome expansion between 10 and 15 Ma, while more southerly savanna has origins that may be no older than 3–4 Ma. Our results also match closely to fossil evidence supporting dates for the first appearance of grassy ecosystems at tropical to subtropical latitudes around this time. In East Africa, earliest evidence of grasslands dates to between 15 and 17.5 Ma [see ref^32^ and citations therein], with a transition to C_4_ dominated grassland from 10 Ma^33^. While pockets of C_4_ grassland may have existed prior to this latter date, there was a general transition towards an increasing proportion of C_4_ to C_3_ grasses from 10 Ma to present^34^, which likely reflected a more extensive shift to C_4_ grasslands across East and Southern Africa^32^.

The large-scale vegetation change from forest to savanna would have triggered major evolutionary shifts in faunal communities and perhaps facilitated hominin evolution of bipedalism (suggesting ground-living *vs*. tree-climbing in apelike hominins), and larger brains, precipitating the development of speech, use of fire, stone tool making, and hunting^35^. Although our analysis provides no direct evidence linking early hominin evolution to the spread of savanna, it is notable that our savanna age estimates match closely to the hominin fossil record (Fig. 2), which shows oldest lineages, with ages going back to 6–7 Ma, near the equator, and younger lineages arising at higher latitudes, as suggested by the fossil deposits in South Africa from about 2 Ma and coincident with the extraordinary recent discovery of at least 15 *Homo naledi* individuals in the Dinaledi Chamber, which, although not yet geologically dated, appear to be younger than 1 Ma based on a phylogenetic ‘morphological clock’^36^.

Cerling et al.^10^ predicted that savannas arose earliest in the tropics and only later at higher latitudes from the photosynthetic response of C_3_ and C_4_ plants to temperature and partial pressure of CO_2_ (*p*CO_2_). With continued decline of *p*CO_2_ from the Late Miocene, C_4_ grass photosynthesis would be favoured over C_3_ plants by lower growing season temperatures in cooler southern latitudes^11^. While there is some support for this pattern, the fossil record does not provide sufficient details to detect evidence for a latitudinal gradient in the spread of savannas to southern Africa^19^. We here infer the age of African savanna by examining the evolutionary splits between tree species that likely diverged in the savanna biome, and derive age estimates broadly consistent with Cerling et al.’s^10^ original prediction on savanna spread.

Phylogenetic data offer a valuable source of information on past environments in Africa that complements that from the fossil record. Fossils provide detailed point information, whereas phylogenetic data are spatially and temporally extensive, but nonetheless rely on accurately dated and identified fossils for calibration. Importantly, however, while we calibrate our phylogeny using the fossil record, the fossil calibrations are not restricted to fossils from Africa but are informed by the global angiosperm fossil record and our Bayesian posterior distribution of node ages simultaneously integrates across multiple calibration points, providing more reliable estimates than any single data point.

Phylogenetic approaches additionally allow us to step through evolutionary time (see ref.^37^), and thus infer time sequence in the latitudinal expansion of savanna, independent from, although consistent with, isotopic evidence. The timing of origin of lineages with particular adaptations, such as the underground lifeform of trees in fire maintained savannas^38^, can also reveal the ecological context within which early hominin evolution played out. A natural extension would be to explore other signature clades, which, when calibrated using different fossils, could provide independent lines of evidence. For example, different elements of the biota characterizing the African savanna (e.g., spiny plants^39^, bovids^40^, carnivores^41^), have diversified since the mid Miocene, contemporaneous with our tree ages. Our focus on trees here provides a broad-brush approach to the origin and spread of savanna. With the addition of better resolved and dated phylogenies, we may gain further insights into our prehistory.

## Methods

### Phylogenetic and spatial data

We focused on the woody plants of sub-Saharan Africa, and sample all the major lineages of southern African trees, following recent taxonomic rearrangements (notably, the quintessentially African genus *Acacia* is now recognised as polyphyletic and African *Acacia* are included in our dataset as two separate genera, *Senegalia* and *Vachellia*, following ref.^42^). Phylogenetic relationships and divergence times were extracted from the dated phylogenetic tree of Charles-Dominique and colleagues^39^, sampling over 1800 of the *ca.* 2000 woody taxa of sub-Saharan Africa. This phylogenetic hypothesis was generated using DNA sequences for the core plant barcodes *matK* and *rbcL* using maximum likelihood (ML) and Bayesian inference. The ML analysis was run in raxmlGUI^43^, enforcing topological constraint based on an APG III backbone in Phylomatic^44^. Branch lengths were made proportional to time using BEAST^45^ assuming an uncorrelated lognormal distribution for the molecular clock model and 20 secondary calibration points obtained from ref.^46^ as minimum age constraints. Full details are provided in refs.^38^ and^39^.

Species range maps were obtained from ref.^38^, and represent outputs from MaxEnt^47^ species distribution models fitted to point data from the African plants database (https://www.ville-ge.ch/cjb/) supplemented by records from the Naturalis Biodiversity Center (https://www.naturalis.nl/) and the Global Biodiversity Information Facility (GBIF; https://www.gbif.org). The MaxEnt presence-absence maps were estimated from the 19 WorldClim^48^ bioclimatic variables using the equal training sensitivity and specificity threshold^49^ and imposing spatial filters to account for geographically restricted ranges, following ref.^50^.

Because our focus here was on the southward expansion of savanna from the tropics and geographical sampling is increasingly sparse as we move to northern latitudes, we truncated species distributions at the equator. We evaluate sensitivity of our analyses by changing this latitudinal threshold to match distribution data from ref.^39^ (see below), but note that more northerly distributions have generally been less well sampled.

Taxa were coded as primarily associated with one of three broad habitat types: savanna, forest and fynbos, using regional floras and expert knowledge (see ref. 38). Savannas were defined as tree–grass mixtures where C_4_ grasses form a near-continuous herbaceous layer which is absent from forest^51^.

### Statistical analysis

It is not straightforward to assess the directionality of an evolutionary split between forest and savanna trees across the forest-savanna boundary without information on the ancestral state; therefore, we identified early lineage diversification events that likely occurred within the savanna biome by considering only splits in which both sister lineages were savanna trees and extracted their divergence times. The phylogenetic positions of savanna sister pairs are shown in Supplementary Figures S6. We assume the sister pair divergence most likely occurred within the habitat type in which they are both present (i.e., savanna species pairs diverged in the savanna biome). The maximum age between sister pairs thus represents a minimum date for the age of the biome. Younger divergences observed within this time frame would not invalidate the age estimate for the biome, indeed we might predict multiple evolutionary splits between taxa that span the age of the biome from its origin to the present day, but only the oldest split provides information on biome age (i.e. the biome must be at least this old for us to observe an evolutionary divergence between taxa restricted to the biome). To evaluate potential bias in sampling, we repeated the process for forest sister pairs. Because the forest habitat type is ancient, we should not detect any strong trend in the age of forest sister pairs with latitude.

We explored the relationship between latitude (mean of the two species latitudinal distribution ranges) and age of splits between savanna trees using quantile linear regression in the R package quantreg^52^. When we had data on the geographic distribution for only one of the species in a sister pair, we used the mean latitudinal position of that species in our model. The quantile linear regression was performed on the top 10% and 5% of oldest ages as the young splits are uninformative about the first spread of savannas (see above). We tested the regression by Markov chain marginal bootstrapping (999 permutations) and used the model coefficients to infer the timing of savanna establishment.

We believe our approach, focussing on divergence times between sister taxa, has multiple strengths: (1) the relative young age of the savanna has typically not provided sufficient time for tree species to radiate within the biome, thus the sample size of such ‘savanna clades’ would be small. While some clades have radiated spectacularly within some biomes in southern Africa, even these remarkable evolutionary events tend to play-out over time spans that may be greater than the age of the savanna biome (e.g. see refs^53^ and^54^). (2) By focussing on sister taxa we avoid the necessity of inferring ancestral states (i.e. biome affinity) for clades, which is not straightforward and often associated with large uncertainty. (3) Our phylogeny, which is the largest reconstructed phylogeny for African woody taxa, is obviously incomplete; woody taxa are not a monophyletic group, and as we move towards the root of the phylogeny clades are more likely to include species that occur outside Africa, and which are therefore not included in our sample. By contrast, we are fairly confident that the sister pairs we identify represent true sister pairs. Nonetheless, we also recognise limitations to a sister-taxon approach. Divergence times between extant sister pairs can be influenced by both speciation and extinction (extinction of a sister taxon can draw back divergence times to the most recent common ancestor of extant taxa). Although for our purposes, here, this distinction is not important unless the probability of a sister taxon going extinct varies both across latitude and between forest and savanna trees. It is also possible that by focussing on sister taxa, we miss information on diversification events deeper in the tree. However, if the signature of savanna-mediated diversification was deeper in the phylogeny, we would not expect to see any latitudinal signature in sister taxa ages, thus, this provides a reasonable null expectation to test in our analysis.

Our analysis additionally assumes that species present-day distributions provides a meaningful approximation of their historical origins. It has been suggested that the paleoclimate of southern Africa was been relatively stable through the Quaternary^55^, with larger climatic shifts, such as those associated with changes in the Benguela Current upwelling system^56^, occurring over the evolutionary time spans of our data, and are likely part of the explanation for the patterns we observe. To explore evolutionary lability in biome affinity, we recoded each biome type as a binary variable, estimated strength of phylogenetic niche conservatism in biome membership using the D-statistic^31^, a measure of phylogenetic signal in a binary trait, and tested significance using permutations (n = 1000).

The geographical distribution of evolutionary split ages was projected by overlaying species distributions on a 0.1° × 0.1° grid, and weighting cells by the mean divergence time of overlapping species. The value of each map cell therefore represents the average evolutionary age of the savanna sisters species with ranges that overlap with it.

## Supplementary information

Supplementary information
