# Supplementary material for: Savanna tree evolutionary ages inform the reconstruction of the paleoenvironment of our hominin ancestors
Source: Sci Rep. 2020 Jul 24;10:12430. doi: 10.1038/s41598-020-69378-0 (PMC7381606; doi:10.1038/s41598-020-69378-0)
Supplement: Supplementary file 1 — Supplementary information [file 41598_2020_69378_MOESM1_ESM.pdf]

Savanna tree evolutionary ages inform the reconstruction of the paleoenvironment of our hominin ancestors

T. Jonathan Davies[1,2], Barnabas H. Daru[3], Bezeng S. Bezeng[1], Tristan Charles-Dominique[4,5], Gareth P. Hempson[6,7], Ronny M. Kabongo[1], Olivier Maurin[8], A. Muthama Muasya[4], Michelle van der Bank[1], & William J. Bond[4,7]

[1]African Centre for DNA Barcoding, Department of Botany and Plant Biotechnology, University of Johannesburg, Johannesburg 2006, South Africa.

[2]Departments of Botany, Forest & Conservation Sciences, University of British Columbia, Biodiversity Research Centre, Vancouver, BC V6T 1Z4, Canada.

[3]Department of Life Sciences, Texas A&M University-Corpus Christi, Corpus Christi, TX 78412, USA.

[4]Department of Biological Sciences, University of Cape Town, Rondebosch 7701, South Africa.

[5]Xishuangbanna Tropical Botanical Garden, Chinese Academy of Sciences, Center for Integrated Conservation, Community Ecology and Conservation, Menglun, 666303 Yunnan, China.

[6]School of Animal, Plant and Environmental Sciences, University of the Witwatersrand, Wits 2050, South Africa.

[7]South African Environmental Observation Network, National Research Foundation, Pretoria 0083, South Africa.

[8]Royal Botanic Gardens, Kew Richmond, Surrey, TW9 3DS, United Kingdom.

Supplementary Table 1. Range of age and localities of African Hominid fossils. FAD is for first appearance datum (earliest fossil evidence); LAD is for the last appearance datum (most recent fossil evidence). We report localities of fossil sites in Africa only. Data are extracted from Wood and Lonnergan (ref. 1).

Taxon name	Fossil name	FAD	LAD	Locality (in Africa)	References
Sahelanthropus tchadensis	Toumai	7	6	Toros-Menalla, Chad;	2,3
Orrorin tugenensis	Millenium Man	6.6	5.7	Baringo, Kenya; Tugen Hills, Kenya	4,5
Ardipithecus kadabba	-	5.8	5.2	Middle Awash, Ethiopia	6,7
Ardipithecus ramidus s.s.	Ardi	4.5	4.3	Middle Awash, Ethiopia; perhaps also Lothagam and Tabarin, Kenya	8,9
Australopithecus anamensis	-	4.5	3.9	Allia Bay and Kanapoi, Kenya	10,11
Australopithecus afarensis s.s.	Lucy	4	3	Laetoli, Tanzania; White Sands, Hadar, Maka, Belohdelie and Fejej, Ethiopia; Allia Bay and West Turkana, Kenya.	12,13
Kenyanthropus platyops	-	3.5	3.3	West Turkana, Kenya; perhaps Allia Bay, Kenya.	14
Australopithecus bahrelghazali	-	3.5	3	Koro Toro, Chad	15
Australopithecus africanus	Taung	3	2.4	Sterkfontein, Makapansgat, Taung and Gladysvale, South Africa	16,17
Australopithecus garhi	-	2.5		Middle Awash, Ethiopia	18
Paranthropus aethiopicus	Black Skull (Omo 18)	2.5	2.3	Shungura Formation, Omo region, Ethiopia; West Turkana, Kenya; Melema, Malawi.	19,20
Paranthropus boisei s.s.	Nutcracker Man	2.3	1.4	Olduvai and Peninj, Tanzania; Omo Shungura Formation and Konso, Ethiopia; Koobi Fora, Chesowanja and West Turkana, Kenya.	21-23
Paranthropus robustus	Eurydice	2	1.5	Blauuwbank Valley, South Africa	24
Homo habilis s.s.	Handy Man	2.4	1.4	Olduvai Gorge, Tanzania; Koobi Fora and perhaps Chemeron, Kenya; Omo (Shungura) and Hadar, Ethiopia, East Africa; perhaps also Sterkfontein, Swartkrans, and Drimolen, South Africa.	25
Homo rudolfensis	-	2.4	1.6	Koobi Fora, and perhaps Chemeron, Kenya; Uraha, Malawi	26-28
Homo ergaster	Turkana Boy	1.9	1.5	Koobi Fora, Kenya	29
Homo erectus s.s.	Kedung Brubus	1.8	0.03	Awash valley Ethiopia; Olduvai Gorge, Tanzania; Thomas Quarry, Morocco	30-31
Homo heidelbergensis	Mauer	0.6	0.1	Kabwe, Zambia; Middle Awash, Ethiopia	32
Homo sapiens s.s.	Human species	0.2	Today	Many sites but earliest in Kibish, Ethiopia	33,34

REFERENCES

1. Wood, B., & Lonergan, N. The hominin fossil record: taxa, grades and clades. *Journal of Anatomy* **212**, 354-376 (2008).
2. Brunet, M. *et al.*, A new hominid from the Upper Miocene of Chad, Central Africa. *Nature* **418**, 145–151 (2002).
3. Vignaud, P. *et al.*, Geology and paleontology of the Upper Miocene Toros-Menalla hominid locality, Chad. *Nature* **418**, 152–155 (2002).
4. Pickford, M. Late Miocene sediments and fossils from the Northern Kenya Rift valley. *Nature* **256**, 279–284 (1975).
5. Senut, B. *et al.*, First hominid from the Miocene (Lukeino Formation, Kenya). *CR Acad Sci, Paris* **332**, 137–144 (2001).
6. Haile-Selassie, Y. Late Miocene hominids from the Middle Awash, Ethiopia. *Nature* **412**, 178–181 (2001).
7. Haile-Selassie, Y. Asfaw, B. & White, T. D. Hominid cranial remains from Upper Pleistocene deposits at Aduma, Middle Awash, Ethiopia. *Am J Phys Anthropol* **123**, 1–10 (2004).
8. White, T. D. Suwa, G. & Asfaw, B.Australopithecus ramidus, a new species of early hominid from Aramis, Ethiopia. *Nature* **371**, 306–312 (1994).
9. White, T. D. Suwa, G. & Asfaw, B. Australopithecus ramidus, a new species of early hominid from Aramis, Ethiopia – a corrigendum. *Nature* **375**, 88 (1995).
10. Patterson, B & Howells, W. W. Hominid humeral fragment from early Pleistocene of northwest Kenya. *Science* **156**, 64–66 (1967).
11. Leakey, M. G., Feibel, C.S., McDougall, I. & Walker, A. New four million year old hominid species from Kanapoi and Allia Bay, Kenya. *Nature* **376**, 565–571 (1995).
12. Kohl-Larsen, L. *Auf den Spuren des Vormenschen: forschungen, Fahrten und Erlebnisse in Deutsch-Ostafrika (Deutshe Afrika-expedition 1934–1936 und 1937–1939* (Strecker und Schröder, Stuttgart, 1943).
13. Johanson, D.C., White, T.D. & Coppens, Y. A new species of the genus Australopithecus (Primates: Hominidae) from the Pliocene of East Africa. *Kirtlandia* **28**, 1–14 (1978).
14. Leakey M. G. *et al.*, New hominin genus from eastern Africa shows diverse middle Pliocene lineages. *Nature* **410**, 433–440 (2001).
15. Brunet, M. *et al.*, Australopithecus bahrelghazali, une nouvelle espece d'Hominide ancien de la region de Koro Toro (Tchad). *CR Acad Sci* **322**, 907–913 (1996).
16. Dart, R. A. Australopithecus africanus: the man-ape of South Africa. *Nature* **115**, 195–199 (1925).
17. Partridge, T. C., Granger, D. E., Caffee, M. W. & Clarke, R. J. Lower Pliocene hominid remains from Sterkfontein. *Science* **300**, 607–612 (2003).
18. Asfaw, B. *et al.*, Australopithecus garhi: a new species of early hominid from Ethiopia. *Science* **284**, 629–635 (1999).
19. Arambourg, C. & Coppens, Y. Decouverte d'un australopithecien nouveau dans les Gisements de L'Omo (Ethiopie). *South Afr J Sci* **64**, 58–59 (1968).
20. Chamberlain, A. T. & Wood, B. A. A reappraisal of the variation in hominid mandibular corpus dimensions. *Am J Phys Anthropol* **66**, 399–403 (1985).
21. Leakey, L. S. B. Recent discoveries at Olduvai Gorge, Tanganyika. *Nature* **181**, 1099–1103 (1958).
22. Leakey, L. S. B. A new fossil skull from Olduvai. *Nature* **184**, 491–493 (1959).
23. Robinson, J. T. The affinities of the new Olduvai australopithecine. *Nature* **186**, 456–458 (1960).
24. Broom, R. The Pleistocene anthropoid apes of South Africa. *Nature* **142**, 377–379 (1938).
25. Leakey L. S. B, Tobias, P. V. & Napier, J. R. A new species of the genus *Homo* from Olduvai Gorge. *Nature* **202**, 7–9 (1964).
26. Leakey, L. S. B. Evidence for an advanced plio-pleistocene hominid from East Rudolf, Kenya. *Nature* **242**, 447-450 (1973).
27. Alexeev, V. *The Origin of the Human Race*. (Progress Publishers, Moscow, 1986).
28. Wood, B. A. Origin and evolution of the genus Homo. *Nature* **355**, 783–790 (1992).

29. Groves, C. P. & Mazák, V. An approach to the taxonomy of the Hominidae: gracile Villafranchian hominids of Africa. *Casopis pro mineralogii a geologii* **20**, 225–247 (1975).
30. Dubois, E. Palaeontologische andrezoekingen op Java. *Versl Mijnw Batavia* **3**, 10–14 (1892).
31. Weidenreich, F. Some problems dealing with ancient man. *Am Anthropol* **42**, 375–383 (1940).
32. Schoetensack, O. *Der Unterkiefer des Homo heidelbergensis aus den Sanden von Mauer bei Heidelberg*. (W. Engelmann, Leipzig, 1908).
33. Linnaeus, C. *Systema Naturae* (Laurentii Salvii, Stockholm, 1758).
34. McDougall, I., Brown, F. H. & Fleagle, J. G. Stratigraphic placement and age of modern humans from Kibish, Ethiopia. *Nature* **433**, 733–736 (2005).

Supplementary Table 2. Woody sister taxa associated with the savanna biome in Africa.

Family	Sister species A	Mean latitude (species A)	Sister species B	Mean latitude (species B)	Divergence time (mya)
Zamiaceae	Encephalartos manikensis	NA	Encephalartos middelburgensis	-21.09	0.33
Zamiaceae	Encephalartos concinnus	NA	Encephalartos longifolius	-30.05	3.28
Dilleniaceae	Tetracera boiviniana	-8.20	Tetracera masuiana	-8.30	4.54
Rubiaceae	Hymenodictyon floribundum	-8.91	Hymenodictyon parvifolium	-11.40	9.03
Rubiaceae	Psychotria pumila	NA	Psychotria kirkii	-12.14	5.48
Rubiaceae	Pavetta radicans	NA	Pavetta eylesii	-25.03	1.91
Rubiaceae	Gardenia subacaulis	NA	Gardenia resiniflua	-11.25	1.21
Rubiaceae	Vangueria macrocalyx	-27.77	Vangueria parvifolia	-25.85	1.06
Rubiaceae	Vangueria madagascariensis	-8.24	Vangueria venosa	NA	1.17
Rubiaceae	Afrocanthium mundianum	-28.20	Afrocanthium lactescens	NA	1.63
Rubiaceae	Pygmaeothamnus chamaedendrum	NA	Pygmaeothamnus zeyheri	-17.75	5.50
Rubiaceae	Catunaregam taylorii	-24.09	Catunaregam swynnertonii	NA	0.75
Rubiaceae	Coptosperma nigrescens	-12.53	Coptosperma supra	NA	0.81
Apocynaceae	Adenium multiflorum	-21.65	Adenium swasicum	NA	6.71
Apocynaceae	Carissa bispinosa	NA	Apocynaceae Carissa	-25.20	1.42
Apocynaceae	Carissa praetermisa	NA	Carissa tetramera	-25.56	2.27
Apocynaceae	Saba comorensis	-7.19	Ancylobothrys capensis	NA	4.64
Loganiaceae	Strychnos pungens	-19.12	Strychnos madagascariensis	-16.30	0.81
Loganiaceae	Strychnos cocculoides	-15.39	Strychnos spinosa	-15.28	1.32
Boraginaceae	Ehretia amoena	-22.96	Ehretia rigida	-25.35	4.27
Acanthaceae	Barleria albostellata	-11.96	Barleria rotundifolia	-24.85	8.30
Bignoniaceae	Markhamia obtusifolia	-15.77	Markhamia zanzibarica	-20.35	3.57
Bignoniaceae	Kigelia africana	-7.22	Stereospermum kunthianum	-8.79	6.26
Bignoniaceae	Rhigozum obovatum	-28.19	Rhigozum zambesiacum	-21.46	3.77
Lamiaceae	Vitex rhemanii	NA	Vitex madiensis	-5.94	1.26

Lamiaceae	Clerodendrum eriophyllum	NA	Clerodendrum glabrum	-20.31	2.17
Solanaceae	Lycium villosum	-25.12	Lycium cinereum	-26.28	1.22
Solanaceae	Solanum lichensteinii	NA	Solanum catombelense	-22.77	0.64
Araliaceae	Cussonia arborea	-8.25	Cussonia spicata	-23.15	0.27
Asteraceae	Tarchonanthus camphoratus	-20.33	Tarchonanthus trilobus	NA	0.58
Asteraceae	Lopholaena coriifolia	-26.79	Asteraceae Lopholaena	-25.67	2.27
Asteraceae	Eumorphia davyi	NA	Phymaspermum acerosum	-28.86	3.92
Ebenaceae	Diospyros lycioides	NA	Ebenaceae Diospyros	-30.78	0.52
Ebenaceae	Euclea natalensis	-24.25	Ebenaceae Euclea	-24.25	0.31
Ebenaceae	Euclea crispa	NA	Euclea divinorum	-19.91	2.73
Olacaceae	Ximenia americana	-12.90	Ximenia caffra	-8.13	4.79
Opiliaceae	Opilia amentacea	-8.49	Opilia Genbank	NA	8.72
Meliaceae	Ekebergia capensis	-15.37	Ekebergia pterophylla	-28.14	2.22
Sapindaceae	Zanha africana	-13.28	Zanha golungensis	-10.01	1.72
Anacardiaceae	Ozoroa longipes	-17.07	Ozoroa nitida	-12.90	0.62
Anacardiaceae	Ozoroa albicans	-23.21	Ozoroa sp	NA	0.49
Anacardiaceae	Searsia dentata	-26.66	Searsia pyroides	-12.28	0.30
Anacardiaceae	Searsia pondoensis	-28.05	Searsia wilmsii	-20.90	0.63
Anacardiaceae	Searsia ciliata	-25.35	Searsia zeyheri	-26.41	1.13
Burseraceae	Commiphora pyracanthoides	-21.95	Commiphora glandulosa	-21.80	1.70
Kirkiaceae	Kirkia acuminata	NA	Kirkia wilmsii	-25.82	1.00
Capparaceae	Capparis tomentosa	-20.46	Capparis sepiaria	-11.56	7.47
Capparaceae	Cadaba kirkii	-11.58	Cadaba termitaria	-22.26	5.79
Capparaceae	Maerua decumbens	-8.83	Maerua parviflora	NA	5.67
Capparaceae	Boscia albitrunca	-22.16	Boscia salicifolia	-13.91	2.37
Salvadoraceae	Salvadora australis	-24.17	Salvadora persica	NA	6.04
Moringaceae	Moringa oleifera	-7.88	Moringa ovalifolia	-20.46	10.68
Malvaceae	Grewia microthyrsa	-24.94	Grewia transzambesica	NA	5.79
Malvaceae	Grewia monticola	-20.90	Grewia hexamita	-24.33	0.35
Malvaceae	Grewia bicolor	-17.15	Grewia flava	-23.04	1.71

Malvaceae	Grewia caffra	-25.03	Grewia flavescens	-21.04	0.40
Malvaceae	Sterculia africana	-15.22	Sterculia rogersii	-24.13	0.74
Malvaceae	Dombeya shupangae	-10.25	Dombeya autumnalis	-15.01	0.45
Malvaceae	Dombeya rotundifolia	NA	Dombeya burgessiae	-21.65	0.62
Dipterocarpaceae	Monotes katangensis	NA	Monotes glaber	-13.82	1.95
Combretaceae	Pteleopsis anisoptera	-12.95	Pteleopsis myrtifolia	-21.35	8.70
Combretaceae	Terminalia mollis	-9.67	Terminalia trichopoda	-14.08	0.89
Combretaceae	Terminalia stuhlmannii	-12.87	Terminalia randii	-12.44	1.34
Combretaceae	Combretum tenuipes	NA	Combretum albopunctatum	-18.18	4.89
Combretaceae	Combretum psidioides	-12.23	Combretaceae Combretum	-12.23	3.45
Combretaceae	Combretum celastroides	-14.14	Combretaceae Combretum	-14.14	2.35
Combretaceae	Combretum mkuzense	-20.50	Combretum erythrophyllum	-24.24	0.52
Combretaceae	Combretum apiculatum	-14.93	Combretum molle	-18.77	2.22
Combretaceae	Combretum adenogonium	-15.01	Combretum padoides	-12.88	6.61
Combretaceae	Combretum collinum	-11.71	Combretaceae Combretum	NA	3.75
Combretaceae	Combretum oxystachium	NA	Combretum zeyheri	-17.77	0.58
Melastomataceae	Dissotis princeps	NA	Heterotis canescens	-11.94	15.31
Myrtaceae	Syzygium guineense	-8.62	Myrtaceae Syzygium	-8.62	0.28
Malpighiaceae	Triaspis hypericoides	-22.27	Malpighiaceae Triaspis	-24.74	0.74
Dichapetalaceae	Dichapetalum deflexum	-8.40	Dichapetalum cymosum	-21.41	3.03
Ochnaceae	Brackenridgea arenaria	-12.93	Brackenridgea zanguebarica	-14.76	7.23
Ochnaceae	Ochna natalitia	-27.12	Ochna angustata	NA	1.15
Hypericaceae	Hypericum revolutum	-2.97	Hypericum roeperianum	-8.58	4.14
Phyllanthaceae	Hymenocardia acida	NA	Hymenocardia ulmoides	-15.18	10.61
Euphorbiaceae	Euphorbia espinosa	-15.76	Euphorbia guerichiana	-21.83	0.92
Euphorbiaceae	Croton menyharthii	-15.75	Croton pseudopulchellus	-17.72	4.73
Phyllanthaceae	Uapaca pilosa	-12.11	Phyllanthaceae Uapaca	-11.09	0.79
Phyllanthaceae	Cleistanthus polystachyus	-7.74	Cleistanthus schlechteri	-18.28	13.61
Phyllanthaceae	Bridelia mollis	-24.36	Bridelia cathartica	-10.59	1.12
Celastraceae	Mystroxylon aethiopicum	-9.21	Celastraceae Mystroxylon	-9.21	0.86

Celastraceae	Salacia rhemanii	NA	Salacia bussei	-9.27	3.02
Celastraceae	Gymnosporia putterlickioides	-13.32	Gymnosporia szyszylowiczii	NA	0.85
Celastraceae	Gymnosporia glaucophylla	-27.73	Gymnosporia capitata	-33.29	0.98
Celastraceae	Gymnosporia maranguensis	-24.99	Gymnosporia oxycarpa	NA	0.99
Celastraceae	Putterlickia pyracantha	-29.03	Putterlickia saxatilis	-25.35	1.29
Celastraceae	Putterlickia retrospinosa	NA	Putterlickia verrucosa	-30.22	0.76
Fabaceae	Vachellia luederitzii	NA	Fabaceae Vachellia	-2.57	0.21
Fabaceae	Vachellia exuvialis	-24.39	Vachellia nebrownii	-21.26	0.75
Fabaceae	Vachellia xanthophloea	-15.94	Vachellia kirkii	-11.75	1.32
Fabaceae	Vachellia farnesiana	NA	Vachellia erioloba	-22.37	9.61
Fabaceae	Senegalia welwitschii	-13.40	Fabaceae Senegalia	-25.69	1.11
Fabaceae	Senegalia goetzei	-13.56	Fabaceae Senegalia	NA	0.23
Fabaceae	Senegalia erubescens	-16.88	Senegalia galpinii	-19.36	1.21
Fabaceae	Senegalia mellifera	-14.73	Senegalia mellifera	-14.73	1.09
Fabaceae	Senegalia senegal	-12.33	Fabaceae Senegalia	-12.33	0.71
Fabaceae	Elephantorrhiza goetzei	-15.35	Elephantorrhiza burkei	-23.33	1.36
Fabaceae	Albizia brevifolia	-19.55	Albizia anthelmintica	-16.34	2.49
Fabaceae	Dichrostachys cinerea	-11.18	Fabaceae Dichrostachys	-11.18	0.95
Fabaceae	Xylia mendoncae	NA	Xylia torreana	-18.84	2.56
Fabaceae	Cassia abbreviata	-13.65	Fabaceae Cassia	-13.65	1.86
Fabaceae	Bolusanthus speciosus	-20.00	Pericopsis angolensis	-11.72	26.69
Fabaceae	Ormocarpum kirkii	-15.15	Ormocarpum trichocarpum	-15.43	3.65
Fabaceae	Dalbergia boehmii	-12.21	Dalbergia melanoxylon	-13.91	8.21
Fabaceae	Pterocarpus rotundifolius	-14.31	Pterocarpus lucens	-14.16	5.06
Fabaceae	Pterocarpus brenanii	-16.29	Pterocarpus rotundifolius	-14.31	1.06
Fabaceae	Philenoptera bussei	-14.77	Philenoptera violacea	-17.28	5.99
Fabaceae	Otholobium polystictum	-27.99	Otholobium wilmsii	-28.05	3.32
Fabaceae	Erythrina madagascariensis	NA	Erythrina zeyheri	-27.56	2.43
Fabaceae	Erythrina baumii	-16.67	Erythrina livingstoniana	-19.37	3.24
Fabaceae	Erythrina sacleuxii	-8.52	Erythrina abyssinica	-11.74	1.92

Fabaceae	Rhynchosia monophylla	-20.83	Eriosema psoraleoides	-11.86	10.30
Fabaceae	Indigofera tinctoria	-11.66	Fabaceae Indigofera	-3.87	0.91
Fabaceae	Brachystegia boehmii	-14.55	Brachystegia longifolia	-11.36	0.80
Fabaceae	Guibourtia conjugata	-21.15	Guibourtia sousae	NA	0.57
Fabaceae	Schotia capitata	-25.22	Schotia afra	-29.96	0.48
Fabaceae	Millettia usaramensis	-11.67	Milletia makoudensis	NA	9.81
Fabaceae	Sophora inhambanensis	-14.93	Sophora tomentosa	-11.50	6.63
Fabaceae	Julbernardia globiflora	-14.08	Julbernardia paniculata	-12.58	2.55
Fabaceae	Copaifera baumiana	-13.38	Baikiaea plurijuga	-16.15	3.53
Fabaceae	Scorodophloeus fischeri	-8.17	Scorodophloeus torrei	-7.13	0.80
Fabaceae	Bauhinia petersiana	-13.85	Bauhinia micrantha	NA	1.04
Rhamnaceae	Berchemia zeyheri	-23.95	Berchemia discolor	-15.26	4.41
Rhamnaceae	Ziziphus zeyheriana	-24.92	Ziziphus abyssinica	-11.58	3.38
Moraceae	Ficus bussei	-13.90	Ficus glumosa	-10.31	0.93
Moraceae	Ficus salicifolia	-21.61	Ficus tettensis	-18.12	1.16
Moraceae	Ficus petersii	NA	Ficus stuhlmannii	-13.96	0.83
Anisophylleaceae	Anisophyllea boehmii	-9.34	Anisophyllea pomifera	NA	1.17
Proteaceae	Protea gaguedi	-14.88	Protea parvula	-27.07	2.06
Proteaceae	Leucospermum gerrardii	-18.48	Leucospermum saxosum	-11.74	0.52
Menispermaceae	Tinospora caffra	-12.61	Tinospora tenera	-16.04	9.80
Xanthorrhoeaceae	Aloe marlothii	-25.37	Aloe excelsa	-17.10	0.55
Arecaceae	Hyphaene coriacea	-14.83	Hyphaene petersiana	-15.29	1.05
Annonaceae	Annona senegalensis	-10.16	Annona stenophylla	-15.51	3.06
Annonaceae	Monodora junodii	-14.47	Annonaceae Monodora	-14.47	0.89

Supplementary Table 3. Maximum age and localities of fossil sites described as containing grassy vegetation (e.g. "rainforest and grassy patches", "grassland", "wooded grassland" etc.)*. Data extracted from Linder (ref. 1).

Site name	Maximum age (millions of years)	Latitude	Hemisphere
Rusinga	18	0.2	S
Bukwa	17.5	1.1	N
Nyakach	15	0.2	S
Fort Ternan	13.7	0.1	S
Tugen Hills	13.3	0.3	S
Lothagam nawata	7.5	2.4	N
Laetoli	4.3	3.1	S
Olduvai	1.9	2.6	S

*presence of grassy vegetation is not necessarily indicative of savanna ecosystems

REFERENCES

1. Linder, H.P. East African Cenozoic vegetation history. *Evolutionary Anthropology* **26**, 300-312 (2017).

Supplementary Figure 1. **Quantile regression of forest tree evolutionary splits across latitudes.** The solid lines indicate the 90th quantile of divergence times between forest trees (see Methods) (slope = 0.08 [s.e. = 0.14], t = 0.55, p = 0.58). Latitude indicates degrees south.

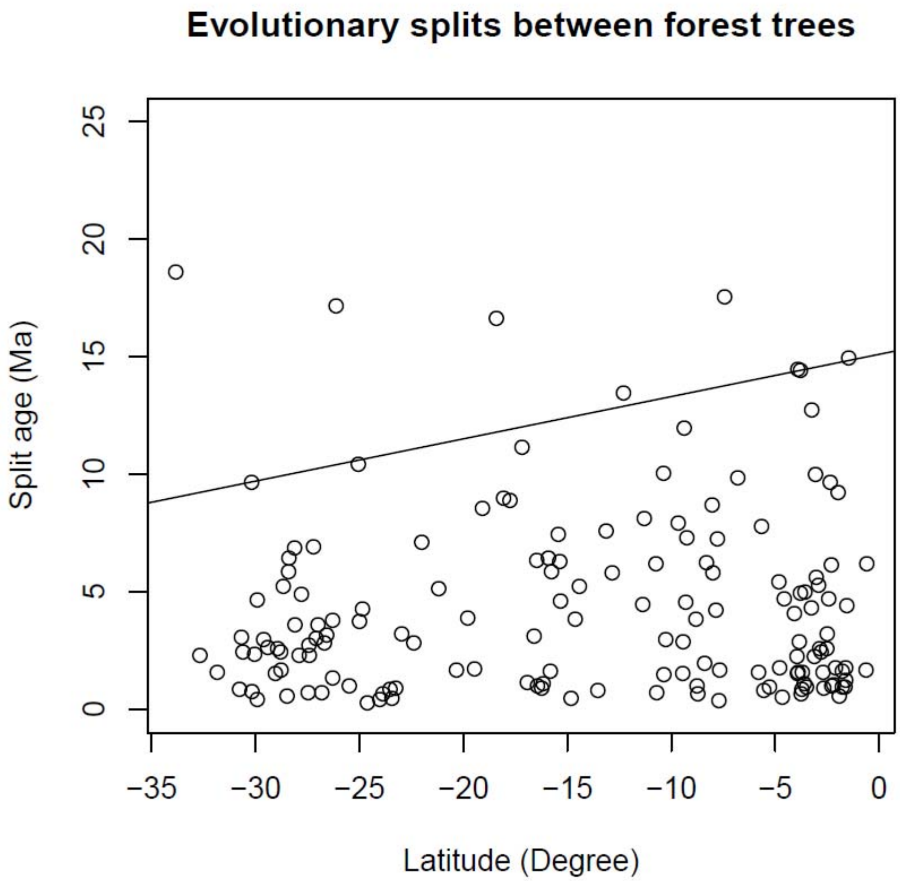

Supplementary Figure 2. **Quantile regression of savanna tree evolutionary splits across latitudes.** The solid line indicated the 90th quantile of divergence times between savanna trees (see Methods). Latitude indicates degrees south, calculating mean latitude from ranges truncated at 15° North and excluding evolutionary splits between sister taxa older than 25 Ma.

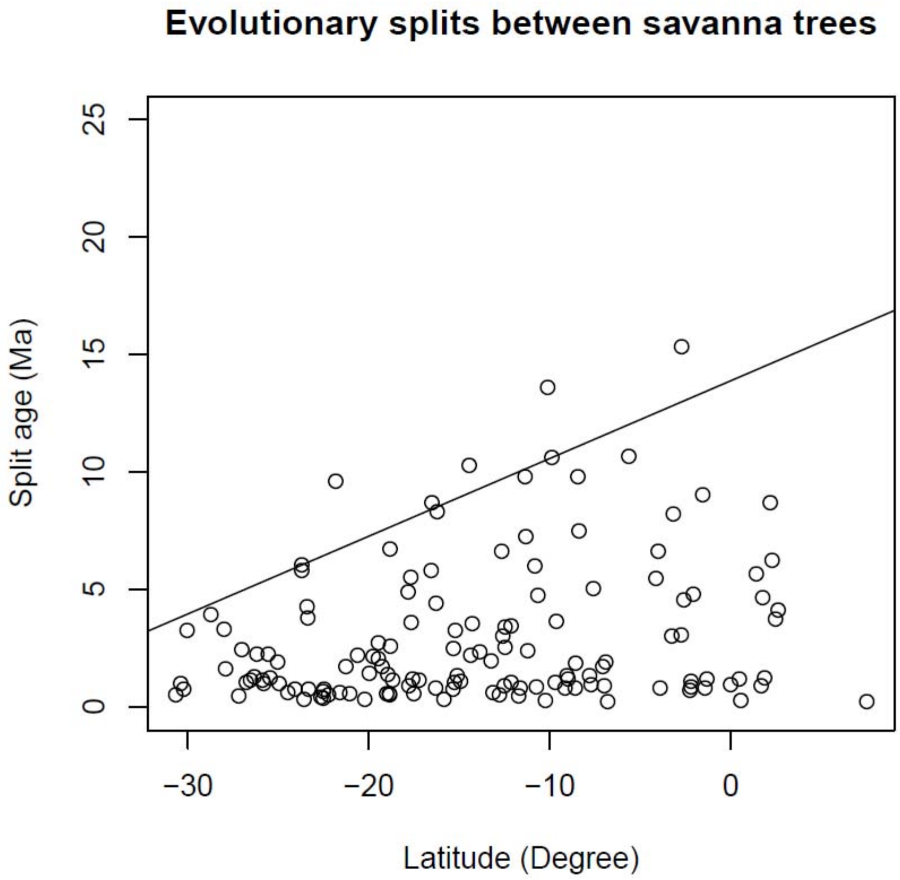

Supplementary Figure 3. **Quantile regression of forest tree evolutionary splits across latitudes.** The solid line indicated the 90th quantile of divergence times between forest trees (see Methods). Latitude indicates degrees south, calculating mean latitude from ranges truncated at 15° North and excluding evolutionary splits between sister taxa older than 25 Ma.

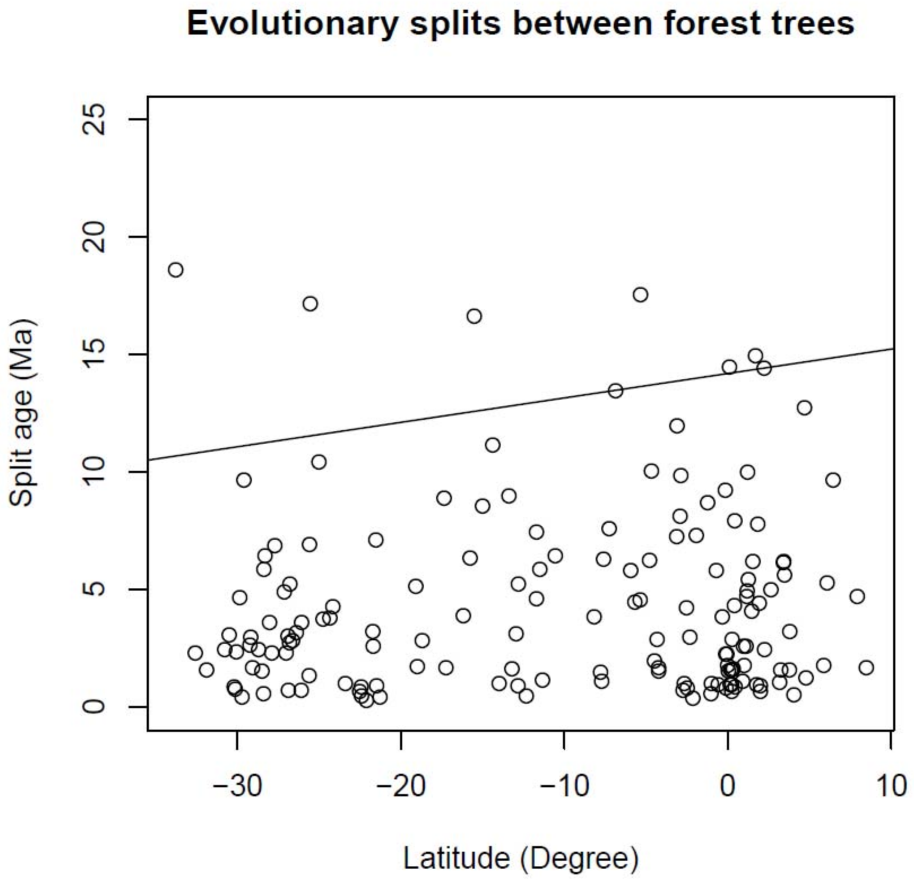

Supplementary Figure 4. **Quantile regression of savanna tree evolutionary splits across latitudes.** Vertical solid lines indicate the 95% Highest Posterior Density (HPD) intervals for divergence times between savanna trees. Fitted lines are through the 10% quantile of the mean HPD (black) upper 95% HPD (red) and lower 5% HPD (blue). Latitude indicates degrees south, with ranges truncated at the equator.

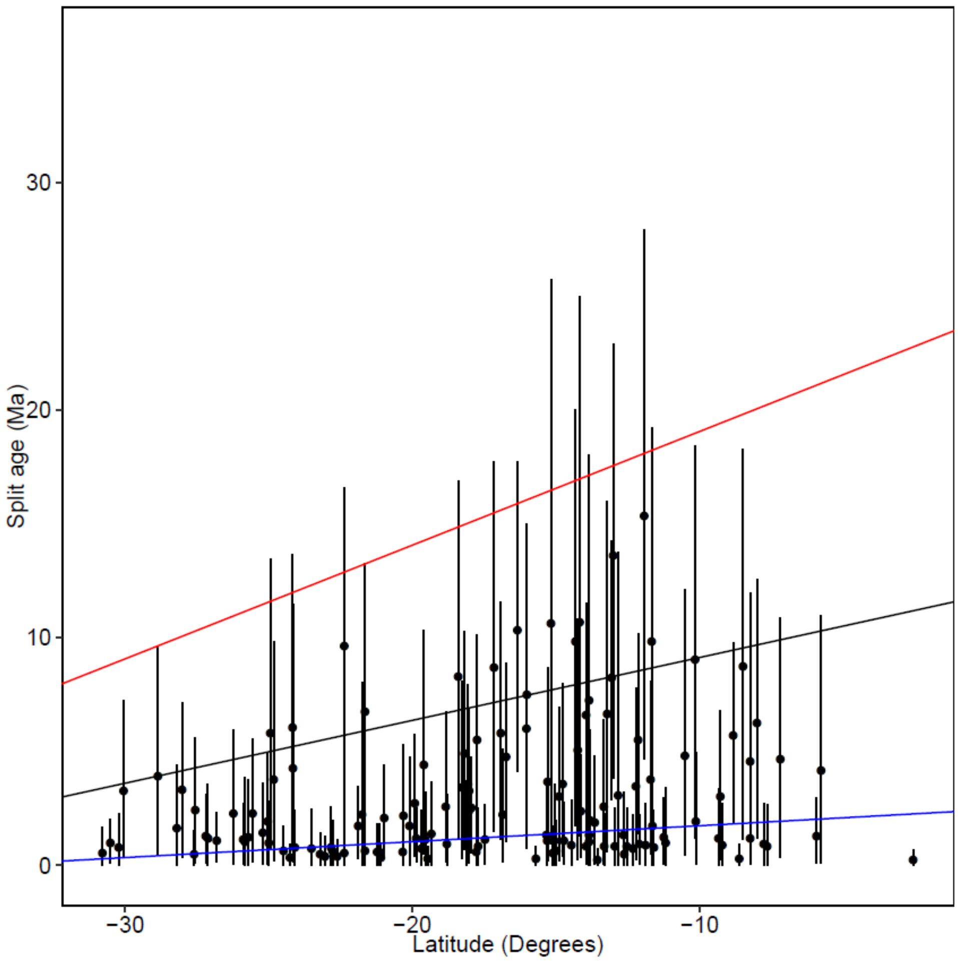

Supplementary Figure 5. **Quantile regression of savanna tree evolutionary splits across latitudes.** Vertical solid lines indicate the 95% Highest Posterior Density (HPD) intervals for divergence times between savanna trees. Fitted lines are through the 10% quantile of the mean HPD (black) upper 95% HPD (red) and lower 5% HPD (blue). Latitude indicates degrees south, with ranges truncated at 15° North.

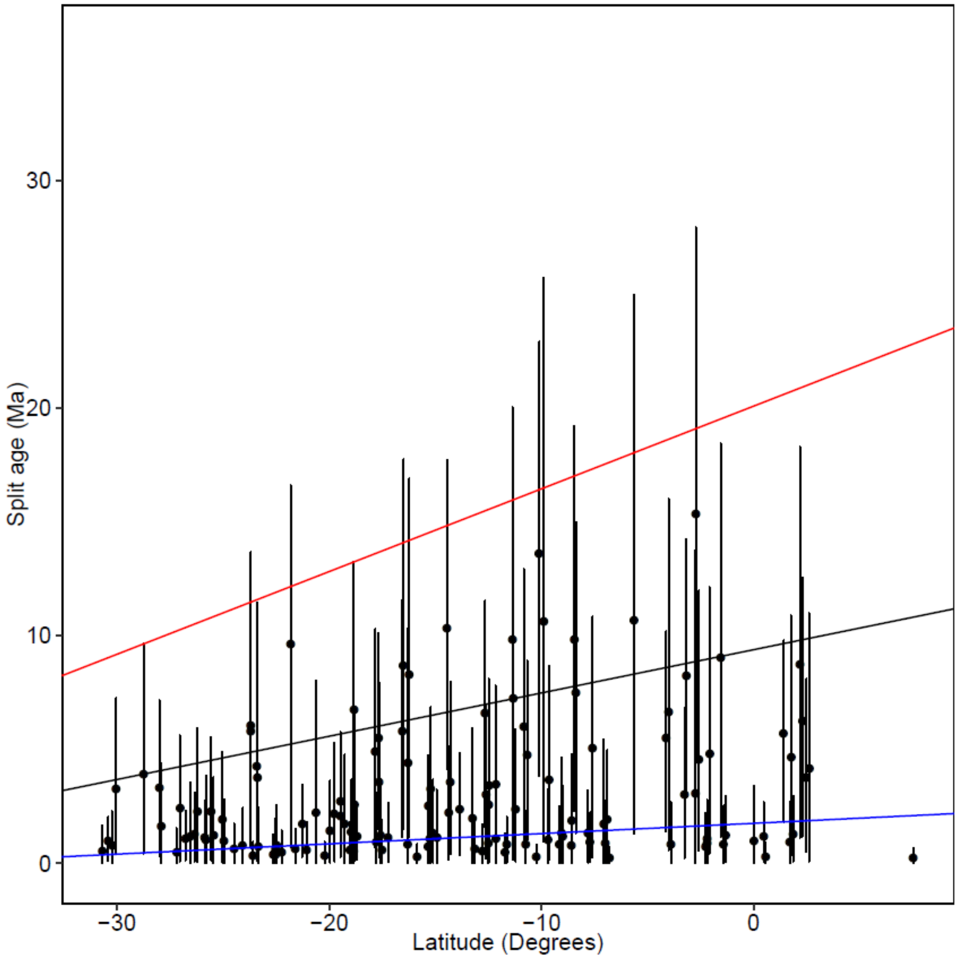

Supplementary Figure 6. **Phylogeny of woody plant taxa for southern Africa.** Branch lengths are scaled to millions of years, and tip labels are coloured to highlight the phylogenetic placement of the savanna sister pairs included in our analyses (teal).

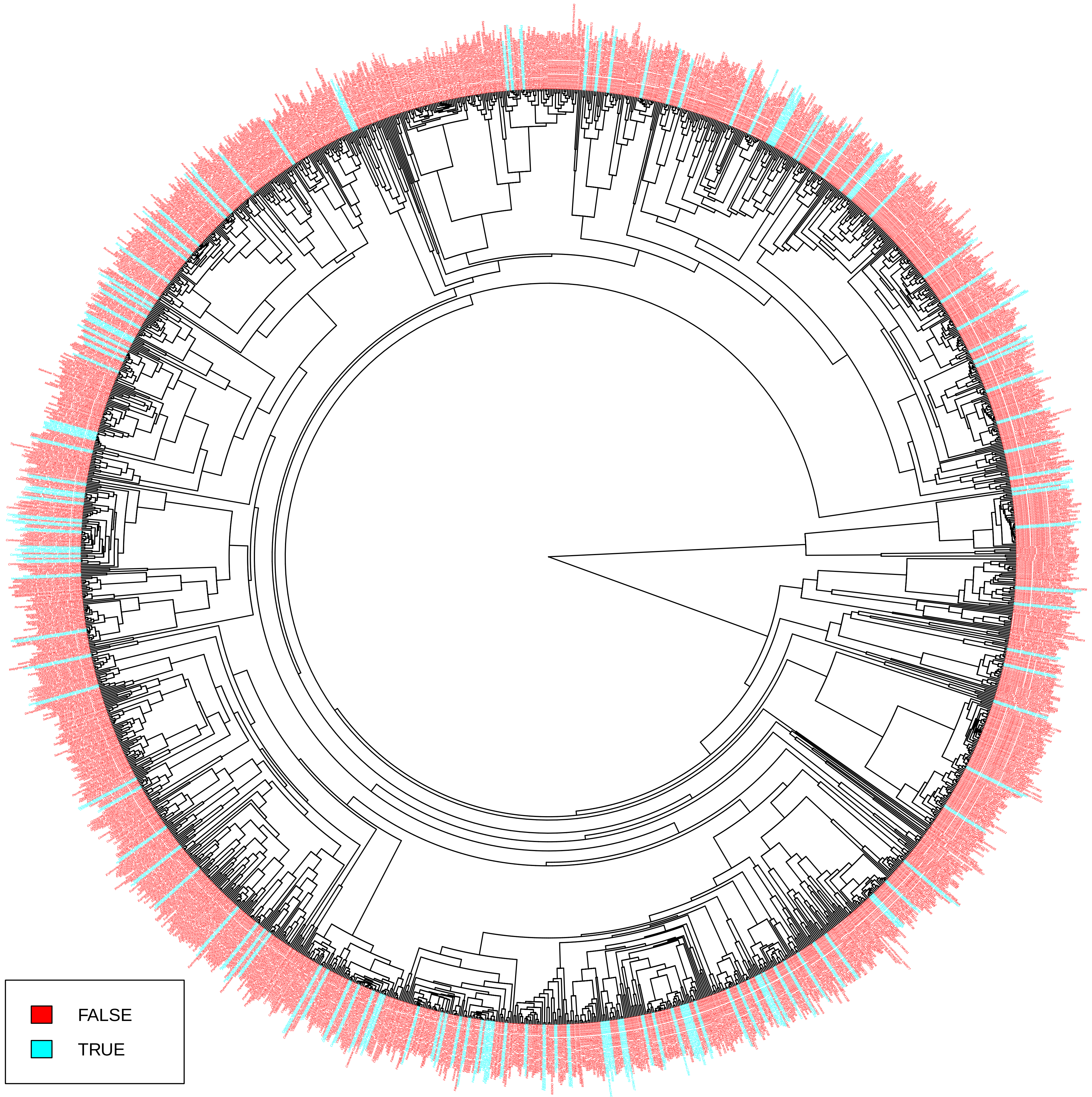
FALSE
TRUE
